# The Extrinsic and Intrinsic Roles of PD-L1 and Its Receptor PD-1: Implications for Immunotherapy Treatment

**DOI:** 10.3389/fimmu.2020.568931

**Published:** 2020-10-21

**Authors:** Katie Hudson, Neil Cross, Nicola Jordan-Mahy, Rebecca Leyland

**Affiliations:** Biomolecular Sciences Research Centre, Sheffield Hallam University, Sheffield, United Kingdom

**Keywords:** programmed death-ligand 1, PD-1/PD-L1-targeted therapy, tumor-intrinsic role, oncology models, novel therapeutic strategies, biomarkers, immunotherapy, PD-1

## Abstract

Programmed death-ligand 1 (PD-L1) is an immune checkpoint inhibitor that binds to its receptor PD-1 expressed by T cells and other immune cells to regulate immune responses; ultimately preventing exacerbated activation and autoimmunity. Many tumors exploit this mechanism by overexpressing PD-L1 which often correlates with poor prognosis. Some tumors have also recently been shown to express PD-1. On tumors, PD-L1 binding to PD-1 on immune cells promotes immune evasion and tumor progression, primarily by inhibition of cytotoxic T lymphocyte effector function. PD-1/PD-L1-targeted therapy has revolutionized the cancer therapy landscape and has become the first-line treatment for some cancers, due to their ability to promote durable anti-tumor immune responses in select patients with advanced cancers. Despite this clinical success, some patients have shown to be unresponsive, hyperprogressive or develop resistance to PD-1/PD-L1-targeted therapy. The exact mechanisms for this are still unclear. This review will discuss the current status of PD-1/PD-L1-targeted therapy, oncogenic expression of PD-L1, the new and emerging tumor-intrinisic roles of PD-L1 and its receptor PD-1 and how they may contribute to tumor progression and immunotherapy responses as shown in different oncology models.

## Introduction

**Figure 1:**
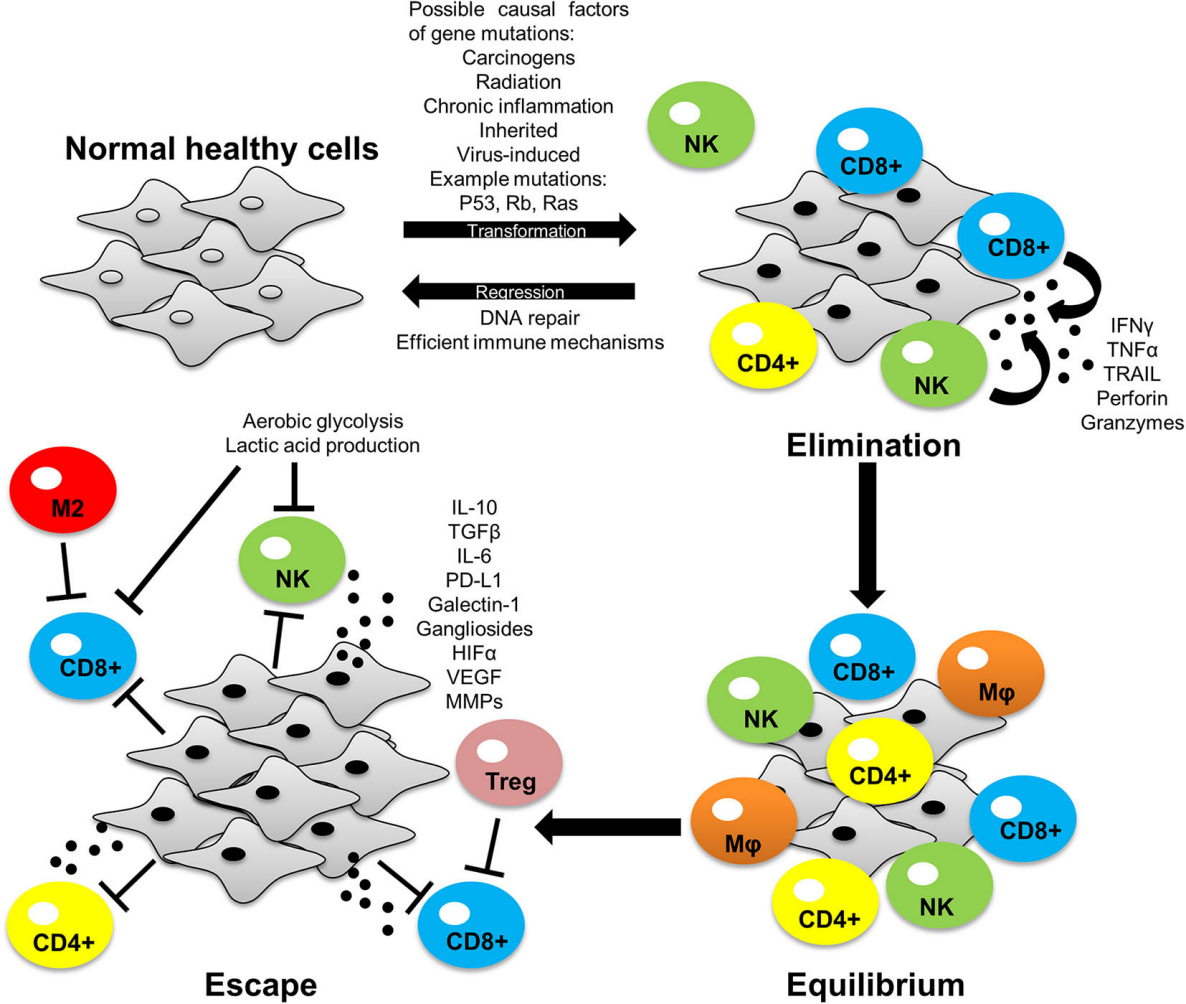
The process of cancer immuno-editing: elimination, equilibrium and escape. Normal healthy cells transform into tumor cells through acquiring mutations that allows for uncontrolled growth of the cell. In the elimination phase, immune cells can recognize and eliminate tumor cells by inducing apoptosis via granule and/or receptor-mediated mechanisms. Some tumor cells avoid immune destruction and enter dynamic equilibrium with immune cells whereby the immune system elicits a potent enough response to contain the tumor cells but not enough to eradicate them. During this phase tumor cells develop increased genetic instability and undergo immune selection, whereby the immune cells eliminate those tumor cells susceptible to immune-mediated killing, whilst selecting those tumor cells with mechanisms to evade the immune system. These selected tumor cells can now proliferate freely and expand leading to immune escape. PD-L1 expression is one of the many mechanisms employed by tumors to facilitate immune evasion and tumor development. Tumor-intrinsic mutations can induce PD-L1 expression and influence tumor cell-immune cell interactions within the tumor microenvironment to favor tumor growth (as discussed later in this review). Interferon gamma (IFN-γ), tumor necrosis factor alpha (TNF-α), tumor necrosis factor-related apoptosis-inducing ligand (TRAIL), interleukin 10 (IL-10), interleukin 6 (IL-6), transforming growth factor beta (TGF-β), hypoxic inducible factor 1/2 alpha (HIF-1/2α), vascular endothelial growth factor (VEGF), matrix metalloproteinases (MMPs).

Cancer immunotherapies work to re-establish immune-mediated tumor eradication (1). Despite the advancements in immunotherapy over the past decade, the interactions of cells within the tumor microenvironment continue to mediate immune evasion and tumor progression (2). The tumor consists of extracellular matrix components and diverse cell populations such as T cells, B cells, natural killer (NK) cells, macrophages, dendritic cells, fibroblasts and endothelial cells (3). Although immune cells such as NK, CD8+, and CD4+ T cells which migrate to the tumor display anti-tumor activity, over time the tumor microenvironment becomes immunosuppressive, favoring the emergence of tumor promoting cells such as regulatory T-cells (T-regs), myeloid derived suppressor cells (MDSCs) and M2 macrophages (1). This is known as the phenomenon cancer immuno-editing which involves three phases: elimination, equilibrium, and escape (Figure 1). Besides tumor cells acquiring the ability to escape immune recognition, they also employ immune-inhibitory mechanisms to evade the immune systems defenses (4). Immuno-editing occurs in patients with advanced cancers and this process can be influenced by therapies targeting immuno-inhibitory proteins (5, 6). One such mechanism is targeting of programmed death-ligand 1 (PD-L1) (7, 8).

PD-L1, also known as B7-H1 and CD274, is an immune checkpoint inhibitor that binds to its receptor PD-1 expressed by T cells, B cells, dendritic cells, and monocytes (7). PD-L1 is expressed by T cells, B cells, NK cells, dendritic cells, macrophages, MDSCs, and many other cell types such as epithelial and endothelial cells (9, 10). The PD-1/PD-L1 signaling axis regulates immune responses to prevent exacerbated activation and autoimmunity (8, 10). Many tumors exploit this mechanism by overexpressing PD-L1 (11–13). Recently, tumors have also been shown to express PD-1 (14, 15). PD-L1 binding to PD-1 on immune cells induces inhibitory responses which in turn can promotes immune evasion and tumor progression.

Elevated expression of PD-L1 on tumors has been reported to strongly correlate with advanced disease state and unfavorable prognosis in melanoma (11), breast (13), gastric (16), ovarian (12), liver (17), kidney (18), pancreatic (19), and bladder (20) cancer. Immunotherapies targeting the PD-1/PD-L1 signaling axis have become the first-line treatment for some cancers due to their ability to promote durable anti-tumor immune responses in select patients with advanced cancers (21–23), leading to their approval by the USA Food and Drug Administration (FDA). Although PD-1/PD-L1-targeted therapies have demonstrated clinical benefits across a broad range of cancers, some patients are unresponsive, hyperprogressive, or develop resistance (24). The objective response rate of anti-PD-L1 monoclonal antibodies alone is ~15% in non-small-cell lung cancer (NSCLC) (21, 25), ~20% in urothelial carcinomas (23, 26) and ~30% in Merkel cell carcinomas (27, 28). Consequently, novel therapeutic strategies are required to enhance patient response rates through combining PD-1/PD-L1-targeted therapy with other immune approaches, small molecule inhibitors, chemotherapy, or other modalities. Patients considered eligible for PD-1/PD-L1-targeted therapy are those that present with PD-L1-positive tumors, circulating PD-1 positive/CD8+ T cells and/or tumors with high mutational burden (23, 29). However, patient tumors that have shown to lack PD-L1 have also responded positively to PD-1/PD-L1-targeted therapy (10, 30), suggesting that either blocking PD-L1 expression on tumors is not required for anti-tumor responses and inhibition of PD-L1 on immune cells alone may be sufficient or that more sensitive approaches to detecting PD-L1 expression on tumors is required. Conversely, some tumors with high PD-L1 expression have shown to be unresponsive to PD-1/PD-L1-targeted therapy (31), likely due to the lack of immune stimulatory cells present in the tumor microenvironment to elicit an effective anti-tumor immune response, but reasons for this remain to be fully elucidated. However, patients that are intrinsically unresponsive to PD-1/PD-L1-targeted therapy can also demonstrate “primary resistance” whereby their tumors display inadequate T cell infiltration, T cell exclusion, impaired IFNγR signaling, and/or local immune suppression (2, 31). Patients that initially respond to PD-1/PD-L1-targeted therapy also show “acquired resistance” whereby their tumors display loss of T cell function and/or disrupted antigen processing and presentation (31, 32). In recent years, approximately 10% of cancer patients have experienced pseudoprogression in response to PD-1/PD-L1-targeted therapy, whereby patients temporally exhibit rapid progression of their condition before responding successfully to treatment (33). On the other hand, some patients have experienced hyperprogression in response to PD-1/PD-L1-targeted therapy, which is characterized by rapid deterioration of their condition upon initialization of treatment without a successful response; giving patients <2 months to live from onset (33). The reasons for pseudoprogression and hyperprogression in response to PD-1/PD-L1-targeted therapy remain speculative and need to be explored. It is also important for clinicians to be able to distinguish between the two types of responses to inform patient selection for therapy. Further insight into the role of PD-L1 and PD-1 in the tumor microenvironment could allow the identification of more appropriate biomarkers predictive of clinical efficacy to PD-1/PD-L1-targeted therapy necessary to ensure patients receive the maximum clinical benefit whilst avoid immune-related adverse effects (24, 29), pseudoprogression and hyperprogression (33).

## Immune Checkpoint Signaling in Cancer

**Figure 2:**
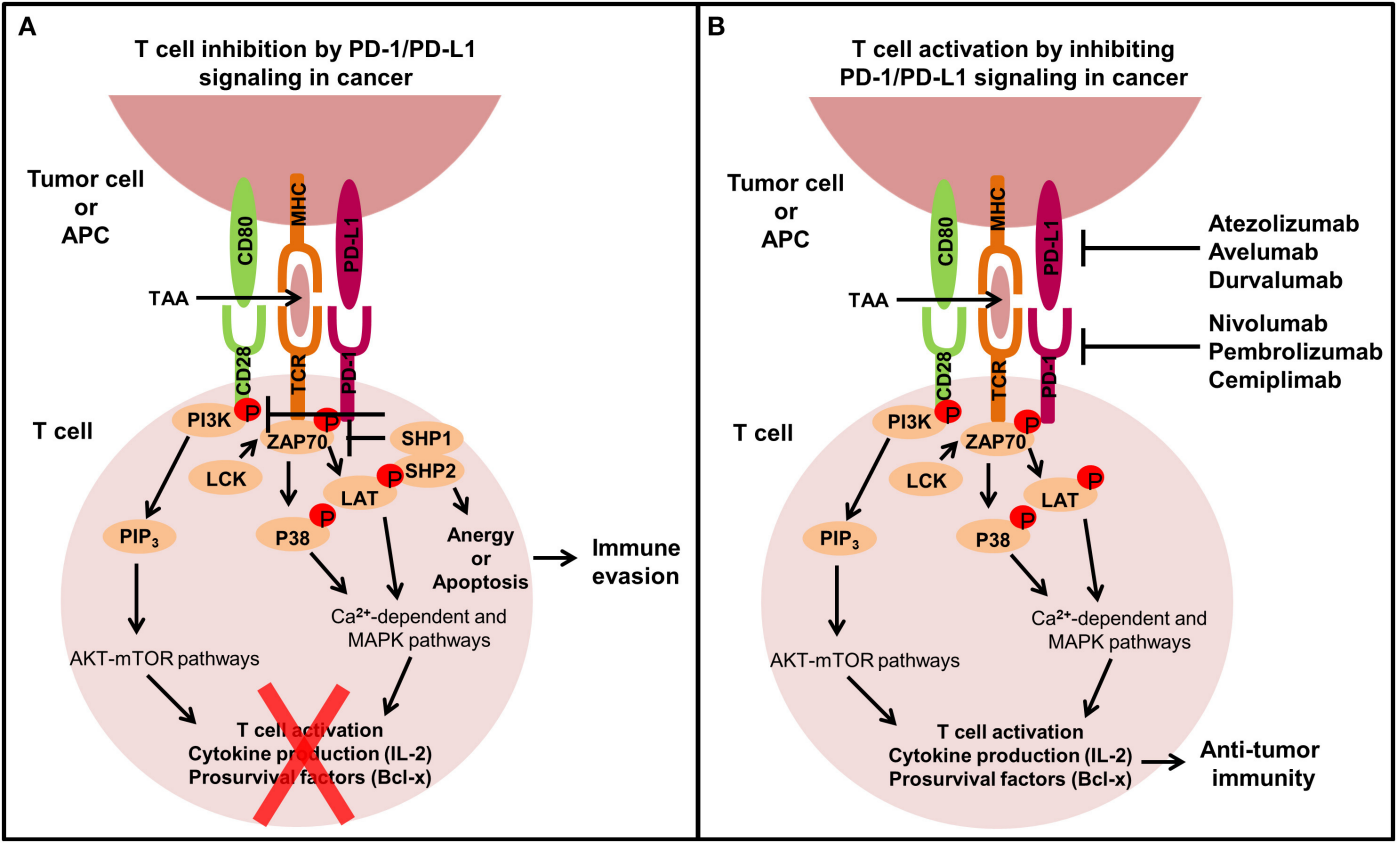
The extrinsic function of the PD-1/PD-L1 signaling axis in cancer. T cells play an important role in modulating immune responses against tumor cells, but tumors can exhibit immune inhibitory mechanisms like overexpressing PD-L1 to avoid T cell-mediated killing. **(A)** When PD-L1 binds to PD-1 expressed on the surface of T cells, T cells become inactivated through the recruitment of SHP1/2 which subsequently inhibits TCR and CD28 co-stimulatory signaling by preventing the phosphorylation of ZAP70 and PI3K, leading to T cell anergy or apoptosis and ultimately immune evasion. **(B)** Monoclonal antibodies targeting the PD-1/PD-L1 signaling axis have been developed to restore immune-mediated eradication of the tumor. PD-1/PD-L1 blockade allows co-stimulatory signal transduction from the TCR and CD28 on T cells upon interaction with APCs or tumor cells. TCR binding to the tumor-associated antigen (TAA) in the MHC complex leads to the phosphorylation of ZAP70, which then phosphorylates P38 and LAT resulting in activation of calcium-dependent and MAPK pathways. Simultaneously, CD80 binding to CD28 phosphorylates PI3K which activates PIP_3_ leading to AKT-mTOR pathway activation. These signaling pathways promote T cell activation, cytokine production and pro-survival factor expression stimulating anti-tumor immunity.

Immune checkpoint molecules expressed on T cells such as cytotoxic T lymphocyte antigen 4 (CTLA-4) and PD-1 regulate immune responses by dampening T cell activation to prevent exacerbated activation and autoimmunity (4, 34). During cancer development anti-tumor immunity is suppressed and immunotherapies targeting CTLA-4 and PD-1 signaling axes have been developed to reactivate T cells to induce immune-mediated tumor eradication (35). Normally, T cell activation requires two signals. Signal one is the T cell receptor (TCR) recognizing and binding to an antigenic peptide presented on an MHC molecule on antigen presenting cells (APCs) or tumor cells. The second is a co-stimulatory signal through CD28 on T cells binding to CD80/CD86 on APCs. CTLA-4 prevents T cell activation by competing with the co-stimulatory molecule CD28 for the CD80/CD86 on APCs (36). Ipilimumab is a CTLA-4 inhibitor approved for the treatment of advanced or unresectable melanoma (36, 37). Unlike CTLA-4 expression restricted to T cells, PD-1 is expressed by activated T cells, B cells and monocytes. PD-1 binds to its two ligands, PD-L1 and PD-L2, expressed primarily by APCs and tumor cells (7). The function of PD-L2 however is not as widely known as PD-L1 (38). Activated PD-1 on T cells through PD-L1 binding counteracts the downstream signaling of the TCR and CD28 co-stimulatory signal by phosphorylating the cytoplasmic immunoreceptor tyrosine-based switch motif leading to the recruitment of Src homology region 2 domain containing phosphatases 1 and 2 (SHP1/2) and slam-associated protein (38, 39). SHP1/2 dephosphorylate the TCR and CD28 proximal signaling molecules including ZAP70 and PI3K, respectively, inhibiting T cell activation, cytokine production and promoting pro-apoptotic molecule expression, ultimately resulting in T cell anergy or apoptosis (2) (Figure 2). The overexpression of PD-L1 in many cancers causes functionally exhausted and unresponsive T cells, promoting immune evasion and tumor progression (7, 11–13) and abrogating PD-L1 expression on tumor cells can enhance sensitivity to T cell killing (40, 41). Signaling of PD-L1 intrinsically within immune cells has not been as well studied as PD-1, however macrophage treatment with anti-PD-L1 antibodies has been shown to upregulate mTOR pathway activity, and RNA-Seq analysis revealed upregulation of multiple macrophage inflammatory pathways (42).

PD-1/PD-L1-targeted therapies yield remarkable anti-tumor immune responses with limited side effects in select patients with advanced cancers (24). They have shown to increase the proliferation of tumor-infiltrating lymphocytes and develop a more clonal TCR repertoire within the T cell population directed against the tumor (32). Currently there are six approved PD-1/PD-L1-targeted therapies for the treatment of multiple cancers as single agents (Table 1); some of which have gained accelerated approval and emerged as front-line treatments for some cancers (35). In 2014, the FDA approved the first anti-PD-1 monoclonal antibody, Nivolumab, for treatment of patients with unresectable or metastatic melanoma and disease progression following Ipilimumab (anti-CTLA-4), based on the CA209037 clinical trial data where Nivolumab alone achieved 31.7% objective response rate (43). Subsequently, Nivolumab was approved for the first-line treatment of metastatic melanoma (44) and second-line treatment for NSCLC (45) and renal cell carcinoma (46) following successful phase I-III clinical trials. Nivolumab has also been approved for classic Hodgkin lymphoma (47), head and neck squamous cell carcinoma (48), bladder cancer (49), and colorectal cancer with microsatellite instability or mismatch repair deficiency (50). Similarly, the anti-PD-1 monoclonal antibody Pembrolizumab is approved for the first-line treatment of metastatic melanoma (51) and NSCLC (25) and second-line treatment for metastatic head and neck squamous cell carcinoma (52) and refractory classical Hodgkin's lymphoma (53). In addition, it has also been approved for gastric/gastroesophageal junction adenocarcinomas (16), cervical cancer (54), and primary mediastinal large B-cell lymphoma (55). Pembrolizumab is also the first therapy to be approved for the treatment of all solid tumors with high mutational burden (56). More recently, Nivolumab and Pembrolizumab have gained accelerated approval for many more cancers (Table 1). Cemiplimab represents a newly approved anti-PD-1 monoclonal antibody for the treatment of metastatic cutaneous squamous cell carcinoma (57). Atezolizumab was the first anti-PD-L1 monoclonal antibody to be approved for treatment of advanced NSCLC and metastatic urothelial carcinoma. Atezolizumab promoted a tolerable and durable objective response rate of 23% and 15% in NSCLC (21, 58) and in metastatic urothelial carcinoma, respectively (22, 59), whilst anti-PD-L1 monoclonal antibodies Avelumab and Durvalumab are approved for the treatment of Merkel cell carcinoma (27, 28) and NSCLC (60), respectively and are both approved for treatment of metastatic urothelial carcinoma (26, 61). Furthermore, these PD-1/PD-L1-targeted therapies are also being investigated for treatment of colorectal, bladder, prostate and breast cancer as well as hematological malignancies and have shown promising results in the early clinical trials.

**Table 1:** FDA approved single agent use of PD-1/PD-L1-targeted therapy for a broad range of cancer types.

**Drug**	**Drug target**	**Study name (Identifier)**	**Population**	**References**
Nivolumab	PD-1	NCT01844505	Metastatic melanoma	(62)
		NCT01642004	Advanced non-small cell lung cancer	(45)
		NCT01668784	Advanced renal-cell carcinoma	(46)
		NCT01592370	Relapsed/refractory classical Hodgkin's lymphoma	(47)
		NCT02488759	Recurrent or metastatic head and neck squamous cell carcinoma	(48)
		NCT02387996	Metastatic urothelial carcinoma	(49)
		NCT02060188	Colorectal cancer with MSI-H and MMR aberrations	(50)
		NCT01658878	Advanced hepatocellular carcinoma	(63)
		NCT01928394	Metastatic small cell lung cancer	(64)
Pembrolizumab	PD-1	NCT01866319	Metastatic melanoma	(51)
		NCT01295827	Advanced non-small cell lung carcinoma	(25)
		NCT02255097	Recurrent or metastatic head and neck cancers	(52)
		NCT02453594	Adults and pediatric patients with refractory classical Hodgkin's lymphoma	(53)
		NCT02335424	Metastatic urothelial carcinoma	(23)
		NCT01295827	Unresectable or metastatic high microsatellite instability or mismatch repair deficient solid tumors	(56)
		NCT02335411	Recurrent locally advanced or metastatic gastric or gastroesophageal junction adenocarcinoma	(16)
		NCT02628067	Recurrent or metastatic cervical cancer	(54)
		NCT02576990	Adults and pediatric patients with refractory or relapsed primary mediastinal large B-cell lymphoma	(55)
		NCT02702414	Advanced hepatocellular carcinoma	(65)
		NCT02267603	Adult and pediatric recurrent locally Merkel cell carcinoma	(66)
		NCT02054806	Advanced small cell lung cancer	(67)
Cemiplimab	PD-1	NCT02760498	Metastatic cutaneous squamous cell carcinoma	(57)
Avelumab	PD-L1	NCT02155647	Merkel cell carcinoma	(28)
		NCT01772004	Metastatic urothelial carcinoma	(61)
Atezolizumab	PD-L1	NCT01375842	Metastatic urothelial carcinoma	(59)
		NCT01903993	Advanced non-small cell lung carcinoma	(21)
Durvalumab	PD-L1	NCT01693562	Advanced urothelial carcinoma	(26)
		NCT02125461	Unresectable stage III non-small cell lung carcinoma	(60)

*PD-1 checkpoint inhibitors currently approved by the FDA include Nivolumab, Pembrolizumab, and Cemiplimab. PD-L1 checkpoint inhibitors currently approved by the FDA include Avelumab, Atezolizumab and Durvalumab. Approved PD-1/PD-L1-targeted therapies are currently under clinical investigation for multiple other cancer types as single agents or in combination with other anti-cancer drugs. Other PD-1/PD-L1-targeted therapies not yet approved by the FDA, such as Pidilizumab targeting PD-1, are also undergoing clinical development for the treatment of multiple cancer types. For each drug displayed in table, the cancer types to which they were approved for treatment are shown in order of approval. MSI-H, High microsatellite instability; MMR, mismatch repair*.

As single agents, immunotherapies targeting the PD-1/PD-L1 signaling axis have demonstrated unprecedented capabilities to elicit anti-tumor immune responses in some patients with advanced cancers (21–23), however the fact remains; there are a large percentage of non-responders or initial responders that acquire resistance (29, 68). Most research associated with PD-L1 and PD-1 has been focused on their extrinsic role to inhibit the immune system, but more recently a tumor-intrinsic role of PD-L1 and PD-1 is emerging in some cancer types; however these roles remain to be fully characterized in all cancers. Important questions to be addressed are the contribution of tumorigenic expression of PD-L1 and PD-1 to intrinsic signaling, whether monoclonal antibodies targeting the PD-1/PD-L1 signaling axis work sufficiently to block this new and emerging role of PD-L1 and PD-1 and whether the intrinsic roles of these proteins are contributing significantly to resistance, relapse to treatment, and hyperprogressive responses in patients.

## Mechanisms Affecting PD-L1 Expression in Tumors

**Figure 3:**
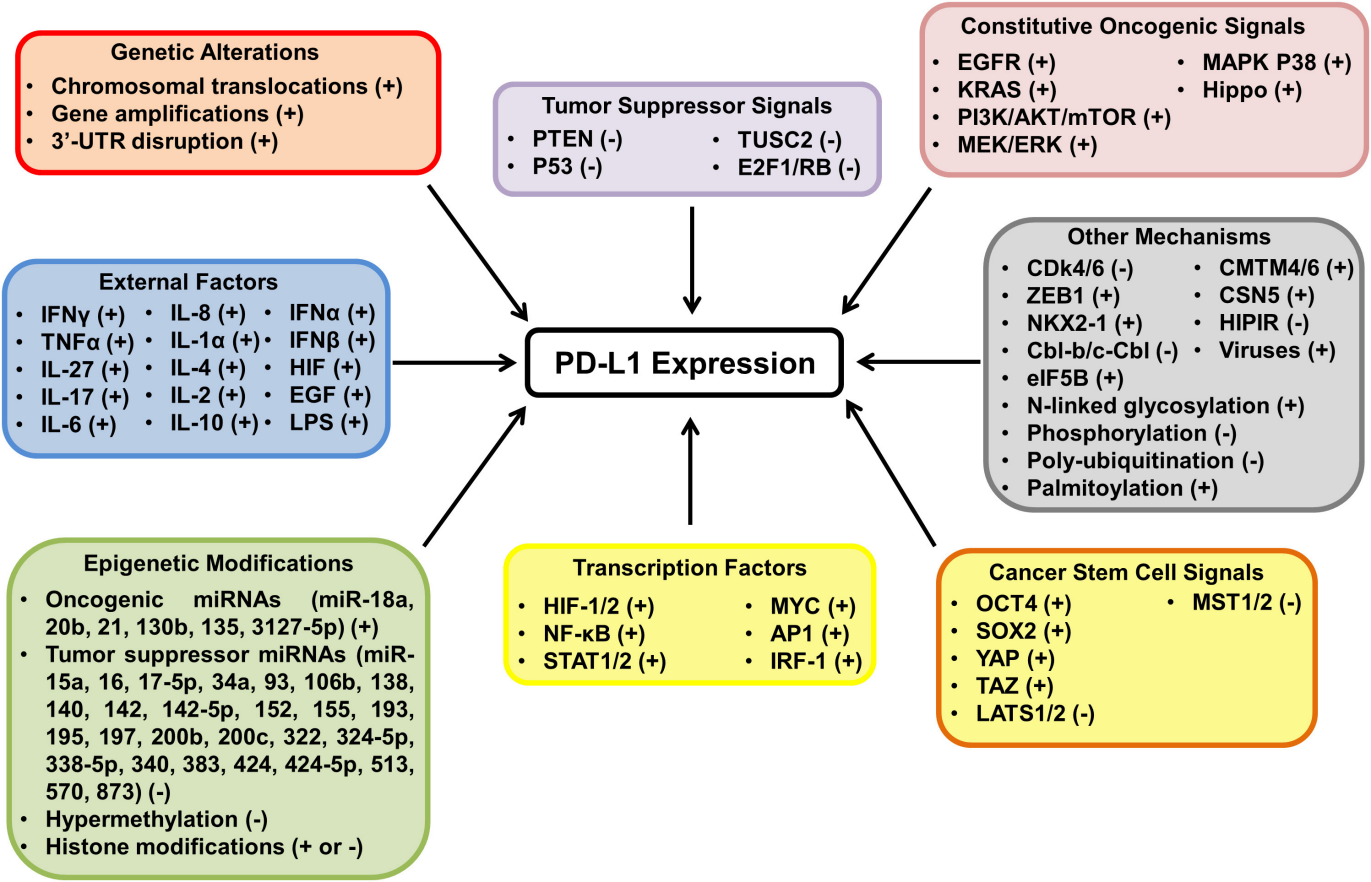
The mechanisms of PD-L1 activation and inactivation in cancer. The diagram highlights the many mechanisms behind PD-L1 regulation in tumor cells and whether the proposed mechanisms have been shown to upregulate (+) or downregulate (−) the expression of PD-L1. PD-L1 expression is regulated at the transcriptional, post transcriptional, translational, and post translational level in tumor cells. Many mechanisms have been shown to modulate PD-L1 expression including genetic aberrations, epigenetic modifications, oncogenic and tumor suppressor signals and extrinsic factors.

The tumor-intrinsic PD-L1 pathway is aberrantly activated in many cancers (11–13, 69). There are several intrinsic and extrinsic mechanisms responsible for PD-L1 regulation in tumor cells, including genetic alterations, epigenetic modifications, oncogenic and tumor suppressor signals, inflammatory cytokines and other factors (Figure 3) (68–80).

### Genetic Aberrations of PD-L1

Several tumors harbor genetic aberrations of the chromosome 9p24.1 which *CD274*, the gene for PD-L1, resides ultimately affecting the expression of PD-L1 (72, 81, 82). Increased copy number alterations on chromosome 9p correlates directly with increased PD-L1 expression (72) and frequently occurs in primary mediastinal B-cell lymphoma (63%) (83), classical Hodgkin lymphoma (40%) (47), triple-negative breast cancer (29%) (84), and soft tissue carcinomas (21.1%) (85). A recent study analyzing 9,771 tumor samples from 22 cancer types revealed a high frequency of copy number gains in bladder, cervical, colorectal, ovarian, and head and neck cancer (more than 15% of tumors), but only a low frequency in pancreatic, renal cell, and papillary thyroid carcinoma (<5% of tumors) (72). In addition, copy number gains are also less frequently observed in gastric cancer (15%) (86), NSCLC (5.3%) (87), small cell lung cancer (1.9%) (88), and diffuse large B-cell lymphoma (3%) (89). PD-L1 copy number gains are associated with substantial therapeutic activity in some cancers due to the high levels of tumor PD-L1 and increased immune infiltrates that they have shown to promote (47, 90). There is some evidence to suggest that PD-L1 chromosomal translocations influence PD-L1 overexpression in certain diffuse large B cell lymphomas (81, 89). Disruption of the 3' untranslated region (UTR) of PD-L1 is another mechanism by which some tumors such as adult T-cell leukemia/lymphoma, diffuse large B-cell lymphoma, and gastric cancer display marked elevation of aberrant PD-L1 transcripts that have become stabilized by truncation of the 3'UTR (69, 82, 91–93). PD-L1 deletions however are more frequently observed in tumors than copy number gains (31 vs. 12%); particularly in melanoma and NSCLC where >50% of tumors harbor PD-L1 deletions (72). PD-L1 deletions, like PD-L1 copy number gains, are associated with a high tumor mutational load and poor prognosis, but the clinical significance of PD-L1 deletions is not yet clear (72, 84).

### Epigenetic Mechanisms Modulate PD-L1 Expression

Epigenetic modifications including microRNAs (miRNAs), promoter DNA methylation, and histone modifications have been shown to modulate PD-L1 expression in different cancers (71, 79, 94–97). A number of miRNAs have been identified to directly or indirectly influence PD-L1 expression (93, 98); the majority of which inhibit PD-L1 expression by tumor cells (Figure 3). One miRNA identified across multiple cancers to inhibit PD-L1 expression is miR-200c which directly binds to the 3'UTR of PD-L1 in hepatocellular carcinoma (99), acute lymphoid leukemia (95), and NSCLC (100). Other miRNAs include: miR-140 (79, 101, 102), miR-142-5p (103, 104), miR-197 (105, 106), miR-34a (95, 107, 108), and miR-424/424-5p (109, 110). miRNAs that positively regulate PD-L1 expression include miR-135 (96) and miR-3127-5p (111) in NSCLC and miR-18a in cervical cancer (79). In colorectal cancer PTEN is directly targeted by miRNAs miR-130b, miR-20b, and miR-21 to indirectly induce PD-L1 expression via PI3K-AKT-mTOR pathway activation (71).

Recently, PD-L1 promoter methylation has been shown to negatively correlate with PD-L1 mRNA and/or protein expression in multiple cancer types including acute myeloid leukemia (78), glioblastoma (112), melanoma (113), head and neck cancer (114), colorectal cancer (115) and prostate cancer (116). The methylation status of the PD-L1 promoter has clinical significance for predicting the outcome of PD-1/PD-L1-targeted therapy (78, 97, 113–116). For example, in NSCLC patients, anti-PD-1 therapy enhanced PD-L1 promoter methylation and reduced PD-L1 expression which mediated resistance to anti-PD-1 immunotherapy Nivolumab in NSCLC patients (117). In addition, histone modifications including methylation and acetylation have been reported to modulate PD-L1 expression in some cancers (118–123). The histone methyltransferase, enhancer of zeste 2 polycomb repressive complex 2 subunit has been shown to suppress PD-L1 expression through mediating trimethylation of the PD-L1 promoter in hepatoma cells (120). Moreover, histone deacetylases have been reported to regulate PD-L1 expression in melanoma cells (122, 124, 125).

### Constitutive Oncogenic Signaling Regulates PD-L1 Expression

Oncogenic and tumor suppressor signaling pathways have been shown to regulate PD-L1 expression (126, 127). Oncogenic signals derived from aberrant receptors, effector molecules and transcription factors leads to the overexpression of PD-L1 by tumors and are associated with poor prognosis and patient response to PD-1/PD-L1-targeted therapy (69, 70, 127, 128). PI3K-AKT-mTOR and RAS-MAPK pathway activation is evidently linked to constitutive PD-L1 regulation in many cancers (69, 129–131). Loss of PTEN (a tumor suppressor that negatively regulates PI3K-AKT-mTOR signaling) or mutations in *PIK3CA* (a catalytic subunit of PI3K) leads to elevated PD-L1 expression via constitutive PI3K-ATK-mTOR pathway activation in squamous cell lung carcinoma (132, 133), NSCLC (130), gliomas (134), colorectal cancer (135), prostate cancer (136), and breast cancer (137). Some tumors harbor mutations in RAS, BRAF, and EGFR and exhibit constitutive RAS-MAPK pathway activation and consequently overexpress PD-L1 (70, 128, 129, 138). BRAF and EGFR mutations correlate with PD-L1 expression, poor prognosis and low patient response to PD-1/PD-L1-targeted therapy in melanoma (70, 138) and NSCLC (128), respectively. Moreover, oncogenic transcription factors including MYC (139), STAT (140), NFκB (141, 142), IRF-1 (143), AP-1 (144), and HIF (145, 146) have been reported to modulate PD-L1 expression at the transcriptional level. MYC expression is found elevated in 70% of cancers (147) and has recently been shown to bind to the PD-L1 promoter transcriptionally inducing PD-L1 expression (148). Similar to MYC, other oncogenic reprogramming factors have been implicated in PD-L1 regulation. OCT4 and SOX2 have both been shown to upregulate PD-L1 expression in cervical cancer (79) and hepatocellular carcinoma (149), respectively, highlighting the necessity of PD-L1 expression for tumor reprogramming functions.

### Extrinsic Factors Promote PD-L1 Expression

Interferon gamma signaling in the tumor microenvironment is primarily responsible for PD-L1 upregulation by tumor cells in most cancer types (76, 150–154). This may be due in part to secretion of IFNγ from tumor specific T-cells within the tumor microenvironment. A study investigating IFNγ-mediated PD-L1 upregulation in multiple cancers including melanoma, renal cell carcinoma, head and neck cancer, and NSCLC, found that IFNγ was able to induce mRNA and protein PD-L1 expression by tumor cells regardless of constitutive PD-L1 expression (76). Although, IFNγ is a dominant driver of PD-L1 expression in various tumors, the mechanism by which IFNγ mediates PD-L1 upregulation appears to be distinct among different cancer types. For example, transcription factors JAK/STAT1, IRF-1 and NFκB are responsible for IFNγ-induced PD-L1 expression in hematopoietic tumors (155), lung cancer (143), and melanoma (141), respectively. IFNγ signaling is often associated with a positive patient response to PD-1/PD-L1-targeted therapy in metastatic melanoma, NSCLC, head and neck cancer, gastric cancer, and urothelial carcinoma (29, 156, 157). Moreover, loss of function mutations in molecules involved in the IFNγ signaling pathway such as JAK1, JAK2, and β2-microglobulin have been identified to render tumor cells unresponsive to IFNγ signaling and mediate intrinsic or acquired resistance to PD-1-targeted therapy (158–160).

Other inflammatory cytokines shown to promote PD-L1 expression by tumor cells include: TNFα in breast (161), prostate, colorectal cancer (162) and hepatocellular carcinoma (152); IL-27 in lung, prostate and ovarian cancer (163); and TGFβ in breast (164) and lung cancer (165). Additionally, some cytokines have been shown to work synergistically to upregulate PD-L1 expression in tumors such as TNFα with IFNγ (166) and with IL-17 (162). Besides inflammatory cytokines extrinsically modulating PD-L1 expression, hypoxia in the tumor microenvironment selectively elevates PD-L1 expression via HIF-1α activation in melanoma, breast, lung, thyroid and prostate cancer (9, 146, 167). In recent studies, HIF-2α has also been shown to correlate with PD-L1 expression in clear cell renal cell carcinoma (168, 169).

Despite the tremendous efforts of scientific researchers to provide insight into the mechanisms behind PD-L1 signal activation in cancer, the regulation of PD-L1 expression by tumors remains to be fully elucidated in all cancer types. Understanding the mechanisms of tumorigenic PD-L1 expression and signaling in different cancer types may provide therapeutic opportunities to alleviate PD-L1-induced intratumoural immunosuppression and overcome resistance to PD-1/PD-L1-targeted therapy. For greater improvement in the efficacy of PD-1/PD-L1-targeted therapy, it is necessary to identify and target tumor-intrinsic mechanisms that are both responsible for controlling PD-L1 expression and promoting tumor progression.

## Tumor-intrinsic PD-L1 Signaling

**Figure 4:**
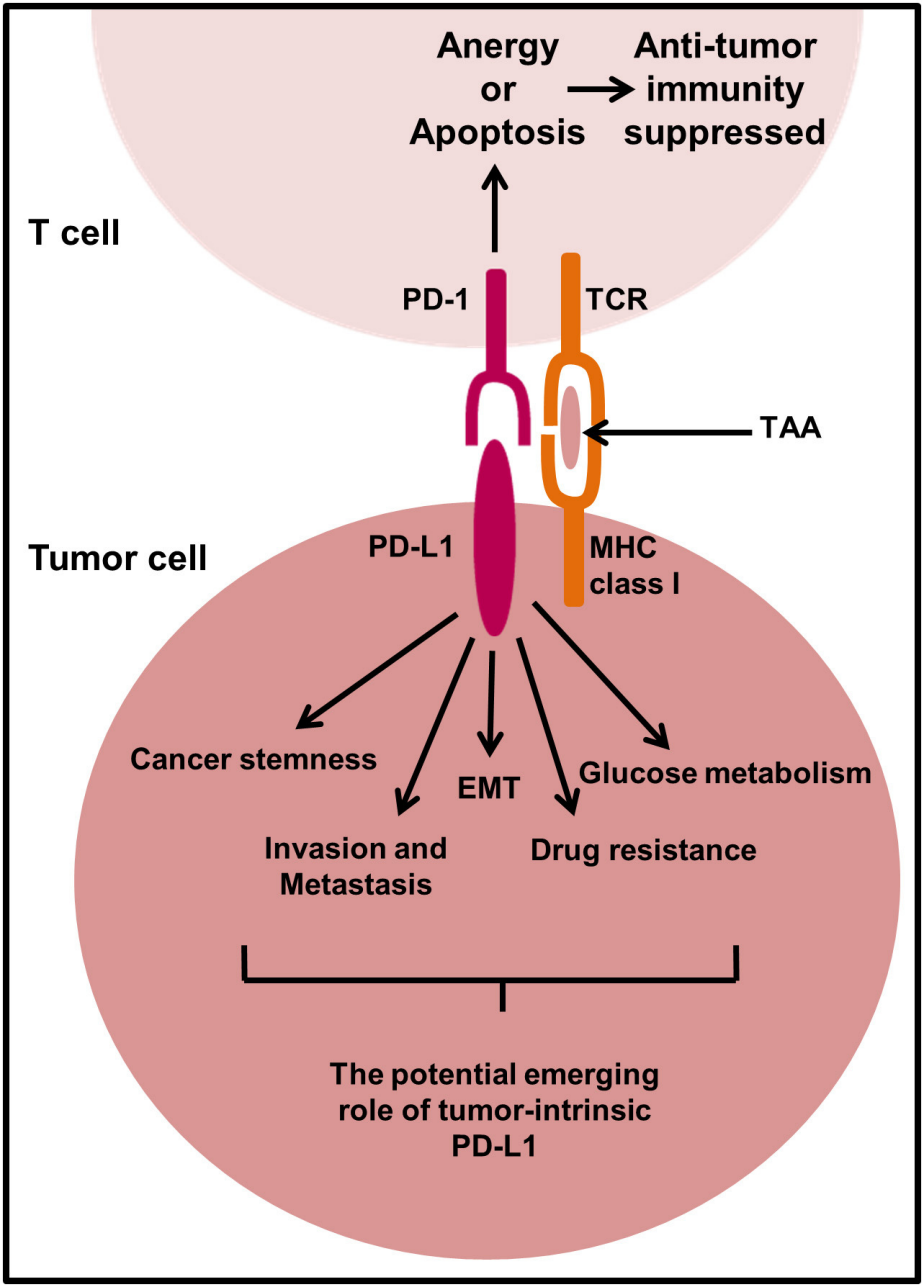
The proposed mechanism of action of PD-L1 in tumor cell signaling. In select cancer types, there is an emerging role of PD-L1 to send pro-survival signals in tumor cells. There is little known about the mechanisms behind PD-L1 signal transduction in tumor cells and more research is required to fully elucidate the potential mechanisms responsible. However, PD-L1 signaling in some tumor cells has been shown to promote cancer initiation, epithelial to mesenchymal transition (EMT), invasion and metastasis, regulate glucose metabolism, and contribute to drug resistance. TAA, Tumor-associated antigen.

To date, there are less than twenty publications investigating the intrinsic role of PD-L1 in tumors; predominantly using RNA interference approaches in two dimensional (2D)-cultured mouse or human cancer cell lines and immunocompromised mouse models. There is an emerging role of PD-L1 to send pro-survival signals within tumor cells to promote cancer initiation, metastasis, development, and resistance to therapy (Figure 4). However, how these emerging pro-survival signals are conveyed intracellularly from cell surface PD-L1 is largely unknown. There is accumulating evidence that intracellular regions of PD-L1 are responsible for transducing survival signals in tumor cells (170–172). Three conserved amino acid sequences including RMLDVEKC, DTSSK, and QFEET motifs have been reported and shown to be located in the intracellular domain of PD-L1. RMLDVEKC and DTSSK motifs were reported to be associated with regulating PD-L1 stability and signal transduction due to the discovery of two specific ubiquitination sites located in the motifs (161, 171). Gato-Cañas et al., demonstrated that the RMLDVEKC motif was required to inhibit IFN-mediated cytotoxicity toward tumor cells via directly preventing STAT3 phosphorylation and caspase-mediated apoptosis. Another study also demonstrated that tumor cells expressing PD-L1 were refractory to Fas- and protein kinase inhibitor Staurosporine-mediated apoptosis (170), which could suggest that the intracellular motifs of PD-L1 may be involved in crosstalk with other signaling pathways; in particular signaling pathways that control tumor cell survival. Other studies have shown that PD-L1 agonists can induce crosslinking between PD-L1 and CD80/CD86 to transduce reverse signaling (173–175). Recently, PD-L1 has been shown to form a heterodimer with CD80, a shared ligand with CTLA-4 and CD28, in *cis* on APCs and tumor cells. This heterodimer was reported to weaken CD80:CTLA4 interaction, but not CD80:CD28 binding indicating that PD-L1 may prevent CTLA-4 inhibitory signals (174, 175). Furthermore, overexpression of CD80 on PD-L1 positive tumor cells was shown to blunt the pro-tumor role of PD-L1 (176). The above studies support the notation that PD-L1 reverse signaling exists in tumor cells. Research efforts should expand on this emerging concept of PD-L1 reverse signaling which has the potential to identify new mechanisms of PD-L1-targeted immunotherapy.

### Tumor-Intrinsic PD-L1 Is Associated With Cancer Initiation

PD-L1 expression has been shown to correlate with the cancer stem cell (CSC)-like characteristics including the expression of CD44 and/or CD133 at high levels on tumor cells. Human head and neck (177), lung (178), and colorectal (179) cancer cells that have CSC-like characteristics (CD44^high^/CD133^high^) were shown to preferentially express PD-L1 compared to CD44^low^/CD133^low^ cancer cells in immunocompromised mouse models either inoculated with a patient-derived xenograft or human cancer cell lines mixed with Matrigel®, respectively. In breast and lung cancer cells CD44 was shown to be a key regulator of PD-L1 expression following shRNA-directed knockdown of CD44 *in vitro* and *in vivo* using a metastatic breast cancer xenograft mouse model (180). Additionally, primary tumor samples from breast and lung cancer patients expressed high levels of PD-L1 correlating with CD44 positivity (180), suggesting that CD44 regulation of PD-L1 expression observed *in vitro* could be similar to that of an *in vivo* human tumor.

OCT4 and Nanog are transcription factors critical for pluripotency and tumorigenesis (98). PD-L1 has been shown to promote OCT4 and Nanog expression via PI3K/AKT pathway in breast CSCs (131). PD-L1 knockdown compromised the capability of breast CSCs to self-renew themselves *in vitro* and *in vivo* using immune deficient nude mice. CSCs ability to self-renew and differentiate into heterogeneous lineages of cancer cells is thought to be responsible for drug resistance and relapse in cancer development and progression (98). A recent study showed that breast cancer stemness is regulated by miR-873 directly suppressing PD-L1 expression and thus PI3K/AKT and ERK1/2 signaling in breast cancer cells, which reduced CSC-like characteristics and enhanced chemosensitivity (181). Tumor PD-L1 has also been shown to promote the tumor-initiating cell generation in immunocompromised murine melanoma and ovarian cancer mouse models; a phenotype which was also verified in a human ovarian cancer cell xenograft mouse model (182, 183). This mechanism of intrinsic PD-L1 to drive tumor stemness was associated with increased mTORC1 signaling (182). However, CSC-like characteristics including high aldehyde dehydrogenase activity, reduced production of reactive oxygen species and a dormant state in the cell cycle were favored following knockdown of PD-L1 in cholangiocarcinoma cell tumors inoculated into mice compared to high PD-L1 expressing tumors (184), indicating that intrinsically PD-L1 may have different roles in different cancer types. Moreover, the CSC-like phenotype is shown to be associated with epithelial to mesenchymal transition (EMT) (98). Chen et al. (100) indirectly knocked down PD-L1 via the microRNA-200/ZEB1 axis in lung adenocarcinoma cells and found that PD-L1 expression correlated with EMT. Low miRNA-200 expressing cells transplanted into a syngeneic immunocompetent mouse model exhibited decreased intratumoural CD8+ T cells and increased metastatic potential due to lack of control over PD-L1 regulation.

### Tumor-Intrinsic PD-L1 and the Promotion of Tumor Growth, Invasion and Metastasis

Besides EMT playing a key role in invasion and metastasis, it has the ability to alter the tumor immune microenvironment to immunosuppressive and influence response to PD-1/PD-L1-targeted therapies (185). PD-L1 knockdown in cultured human gastric cancer cell lines SGC-7901 and AGS reduced cell proliferation, migration, invasion and induced cell cycle arrest *in vitro* and reduced tumor growth and EMT phenotypic marker expression in immunocompromised mice *in vivo* compared to gastric tumors expressing PD-L1 (186). Similarly, in cultured human Jurkat lymphoid leukemia cells and Raji lymphoma cells, PD-L1 knockdown by lentiviral transduction reduced their invasive ability via downregulation of extracellular matrix-degrading enzymes, matrix metalloproteinase 2 and 9 (187). PD-L1 silencing in murine B16 melanoma cells has also been shown to slow tumor growth and reduce metastases to the lungs of immunocompetent mice as well as immunodeficient mice via mechanisms that increase autophagy and reduce mTORC1 signaling (188). These findings may be linked to the intrinsic functions of PD-L1 to promote tumor stemness via mTORC1 signaling (182, 183). Tumor-initiating cells induced by intrinsic PD-L1 signaling are likely to show higher metastatic potential due to their self-renewal capabilities. Interestingly, the same therapeutic effect to reduce lung metastasis was absent in murine ovarian ID8agg cancer cells lacking PD-L1, in immunocompromised mice (188), suggesting the effects of intrinsic PD-L1 may be tumor specific and warrants further investigation. A recent study which knocked down PD-L1 in NCI-H1299 and Calu-1 cells showed enhanced proliferation in comparison to control cells, suggesting a tumor suppressor role of PD-L1 (15). Indeed, PD-L1 expression has been shown to correlate with EMT markers in many solid tumors including gastric, lung, breast, colon, and other common cancers (185, 189). With consideration co-targeting of EMT vulnerabilities and PD-1/PD-L1 signaling axis may have the potential to improve clinical efficacy of immunotherapy by limiting the shift of the tumor microenvironment from immunostimulatory to immunosuppressive during tumor development.

### Tumor-Intrinsic PD-L1 and Regulation of Metabolic Processes

Within the tumor microenvironment, nutrient competition between tumor cells and immune cells may regulate tumor progression and PD-L1 has been reported to directly regulate the metabolism of several cancer cell lines (190, 191). Lactate derived from tumors can suppress the function of T cells by disrupting aerobic glycolysis, a process required for optimal T cell function (190). It has been reported that checkpoint blockade could induce an increase in the glucose concentration within a progressive tumor mouse model, which correlated with glycolytic capacity in tumor infiltrating lymphocytes and increased IFNγ production (191). Interestingly, treatment of B16 melanoma, MC38 colon cancer and sarcoma cancer cell lines *in vitro* with anti-PD-L1 antibodies was shown to reduce aerobic glycolysis mechanisms, including reduced glycolysis enzymes and Akt phosphorylation, indicating a tumor intrinsic role for PD-L1 in enhancing tumor glycolysis. The same results were achieved by shRNA mediated knockdown of PD-L1 (191), strongly suggesting that PD-L1 itself was the modulator of glycolysis in cancer cells. Hypoxic inducible factor, HIF-1α is a well-known modulator of glycolysis in cancer cells (192). The reduced glycolytic activity of cancer cells caused by PD-L1 blockade would subsequently induce an adaptive hypoxic response and stimulate the production of HIF-1α. HIF-1α also directly modulates immune cell activity in the tumor microenvironment to favor tumor growth and induces PD-L1 expression on tumor cells and immune cells; indirectly mediating immune escape and tumor progression (9). Under hypoxic conditions PD-L1 expression was directly induced by HIF-1α on MDSCs in B16-F10 tumor-bearing mice, and PD-L1 blockade increased MDSC-mediated T cell activation by downregulating IL-10 and IL-6 expression (193). Dual blockade of PD-L1 and HIF-1α could further reduce the glycolytic activity of cancer cells caused by PD-L1 blockade and enhance anti-tumor immunity, ultimately leading to cancer cell death.

### Tumor-Intrinsic PD-L1 Facilitates Resistance to Anti-Cancer Therapies

PD-L1 exhibits an anti-apoptotic role in MDA-MB-231 breast cancer cells and silencing PD-L1 in these cells increased cancer cell apoptosis and enhanced cancer cell susceptibility to doxorubicin-induced apoptosis *in vitro* and *in vivo* (194), suggesting that PD-L1 not only prevents cancer cell apoptosis, but also promotes chemotherapy resistance. Likewise, CRISPR/Cas9 knockout of PD-L1 enhanced the sensitivity of human osteosarcoma KHOS and MNNG/HOS cells to doxorubicin and paclitaxel and compromised their ability to form three-dimensional (3D) spheroids *in vitro* (195). Further characterization of the role of PD-L1 in chemotherapy resistance in MDA-MB-231 breast cancer cells discovered that PD-L1 knockdown suppresses the expression of multidrug resistance 1/P-glycoprotein (MDR1/P-gp) via PI3K/AKT pathway *in vitro* (196); recognizing this has an additional therapeutic target. In fact, PD-1/PD-L1 interaction increased survival of breast cancer cells when exposed to doxorubicin (196), suggesting that PD-1/PD-L1-targeted therapy may increase chemotherapy efficacy by inhibiting MDR1/P-gp expression which usually confers resistance in breast cancer cells. Moreover, through culturing of breast (MDA-MB-231 and 4T1) and prostate (DU145) cancer cell lines with recombinant PD-1 or Jurkat T cells it has been shown how PD-1/PD-L1 interactions results in increased resistance to doxorubicin and docetaxel (197). Subsequent knockdown or blockade of PD-1 restored tumor cell chemo-sensitivity and reduced their metastatic potential in a synergistic breast cancer mouse model, suggesting blockade of intrinsic pathways is beneficial for therapy. Conversely, human colorectal cancer cells harboring a *BRAF*^*V*600*E*^ mutation showed that the depletion of PD-L1 suppresses chemotherapy-induced apoptosis through the down regulation of BIM and BIK BH3-only proteins (198), even though depletion alone reduced tumor growth. The effect of PD-L1 on chemosensitivity was confirmed in *BRAF*^*V*600*E*^ mutant MC38 murine tumor xenografts, where PD-L1 knockout cells were less sensitive to chemotherapy due to the suppression of pro-apoptotic molecules, BIM and BIK, compared to parental cells expressing PD-L1. This study highlights the importance of understanding the role of PD-L1 in each cancer type and its subtypes to design effective treatment regimens that will benefit cancer patients.

The tumor-intrinsic role of PD-L1 appears to be similar across all cancer types investigated in the literature to date, with the exceptions of cholangiocarcinoma and contradictory evidence in lung cancer, in that PD-L1 promotes tumor growth and development. However, the molecular mechanisms of PD-L1 exerting pro-tumor activity appear to be distinct amongst different cancer types. Notably, in the studies that have investigated the intrinsic role of PD-L1 in lung cancer the cells utilized were mesenchymal lung cancer cell lines which harbored KRAS and/or p53 mutations, suggesting that the tumor cells metastatic capacity and mutational status may not be determining factors as to whether PD-L1 exhibits a pro-tumor or anti-tumor role in lung cancer. Furthermore, Wang et al. (15) reported that PD-L1 expression reduced lung cancer cell proliferation, suggesting that although PD-L1 expression may limit tumor cell proliferation it is still affecting other tumor characteristics that influence tumor progression. The reasons behind this potential role of PD-L1 in lung cancer warrants further investigation.

## Tumor-Intrinsic PD-1 Signaling

**Figure 5:**
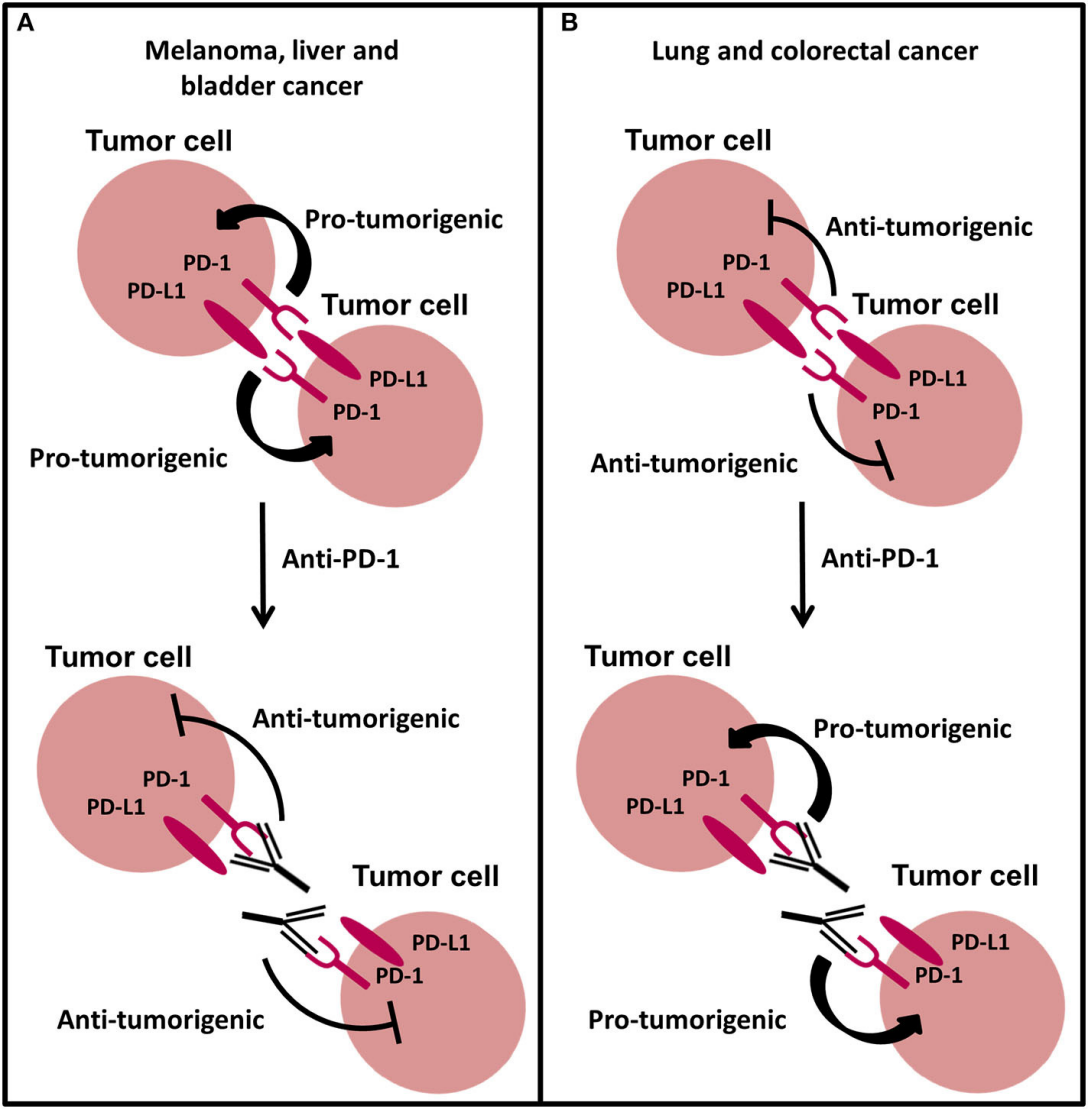
The new and emerging role of PD-1 signaling in cancer. **(A)** Intrinsic PD-1 signaling has been shown to promote tumorigenesis in melanoma, liver, and bladder cancer cells. Anti-PD-1 therapy abrogates this effect inhibiting tumor growth. **(B)** Intrinsic PD-1 signaling in NSCLC and colorectal cancer cells has been shown to inhibit tumorigenesis. Anti-PD-1 therapy preventing PD-1 signaling promotes tumor progression in NSCLC and colorectal cancer cells.

Similar to PD-L1, the expression of PD-1 on T cells and its role to inhibit the immune system is well characterized, but recent studies have found intrinsic expression of PD-1 in tumor cells including melanoma (199), hepatic carcinoma cells (200), ovarian (201), bladder (201), lung (15, 202), and colorectal (15) cancer cells. In melanoma B16 tumors, a subpopulation of PD-1 expressing cancer cells were identified to modulate downstream mTOR signaling and promote tumorigenesis independent of adaptive immunity, in an *in vivo* mouse model lacking an adaptive immune system (Figure 5A) (199). This effect was abrogated with anti-PD-1 therapy, tumor-specific PD-1 knockdown and mutagenesis of intracellular signaling motifs downstream of PD-1, strongly suggesting an intrinsic function of PD-1 to promote tumorigenesis in melanoma. Similar to intrinsic PD-1 in melanoma cells, intrinsic PD-1 in liver cancer cells has been reported to mediate tumorigenesis in immunocompromised mice via regulating mTOR signaling (Figure 5A) (200) and thus combined inhibition of PD-1 and mTOR may be a potential therapeutic strategy for melanoma and liver cancer. Moreover, anti-PD-1 therapy has been reported to reduce the cell growth of bladder RT4 cancer cells cultured in 2D in the absence of adaptive immunity (201), implying that PD-1 expression is potentially oncogenic. Interestingly in murine NSCLC M109 cells, intrinsic PD-1 exhibited an anti-tumor role in immunocompromised mice and when NSCLC cells were treated with anti-PD-1 therapy they demonstrated increased proliferation and tumor growth (Figure 5B) (202). Consistent with this, silencing of PD-1 or therapeutic antibody blockade of PD-1 on the surface of NSCLC and colorectal cancer cells increased proliferation *in vitro* via activating PI3K and MAPK pathways (15), suggesting that PD-1 could be involved in development of resistance to immunotherapy blockade in NSCLC and could provide one explanation for why patients with NSCLC can display hyperprogressive disease following treatment with anti-PD-1 therapy (15, 203). The latter findings also suggest that the tumor suppressor role of PD-1 on cancer cells may not be limited to NSCLC. Although, Wang et al., demonstrated that PI3K and MAPK pathways were activated following anti-PD-1 therapy in NSCLC cells *in vitro* and *in vivo*, their study also showed that PD-1/PD-L1 dysfunction did not activate mTOR, illustrating that the mechanism behind tumor-intrinsic PD-1 to either induce or inhibit tumor growth may be different. However, mTOR activation has been shown to occur in the only two studies investigating tumor-intrinsic PD-1 where PD-1 has a pro-tumor role, which may suggest that mTOR signal activation is necessary for PD-1 to exhibit tumorigenic activity. Therefore, the molecular mechanism behind tumor-intrinsic PD-1 needs to be elucidated in other cancer types to confirm this potential role of mTOR in PD-1 signaling in tumor cells. Furthermore, studies investigating the role of tumor-intrinsic PD-1 in tumors have utilized tumor cell lines that exhibit invasive and metastatic potential. The metastatic potential of cells does not seem to be a factor in determining whether PD-1 is pro- or anti-tumorigenic and nor is it associated with enhanced tumor-intrinsic PD-1 activity (199). Additionally, studies have used both poorly- and well-differentiated tumor cells which have been shown to have the same PD-1-intrinsic function, implying that the differentiated state of the cell is also not a contributing factor to the role PD-1 in tumors. Yao et al., reanalyzed cancer transcriptomic and proteomic data from The Cancer Genomic Atlas Project and The Cancer Cell Line Encyclopedia Dataset to find that tumor-intrinsic PD-1 expression is widespread in many cancer types. This heterogeneity may explain the differential therapeutic effects of anti-PD-1 drugs and could provide crucial information required when selecting suitable patients for treatment dependent on the cancer cell type. However, further work in different cancers and tumor models could also shed more light into this area.

So far, most evidence for PD-L1 and PD-1 signaling in cancer cells is based on 2D cell culture models using murine and human cancer cells and immunodeficient mouse models that can fail to fully recapitulate the human *in vivo* tumor (24, 204). Therefore, more relevant models capable of recapitulating the heterogeneity of the tumor microenvironment during *in vivo* conditions could allow further predictive *in vitro* evaluation of the tumor-intrinsic role of PD-L1 and PD-1, and how these roles may be affected by immunotherapy treatment and influence immune cell function.

## Immunotherapy Blockade of Intrinsic PD-L1 and PD-1 Signaling

Recent reports discussed above suggest that the emerging intrinsic role of PD-L1 is largely pro-tumorigenic in a number of cancers, but that in lung cancer and cholangiocarcinoma, it may act as a tumor suppressor gene. Likewise, the new emerging tumor intrinsic role of PD-1 has also been reported to have differential roles in different cancer cell types and this remains to be further investigated. However, there are currently a limited number of reports investigating how immunotherapeutic drugs potentially modulate these intrinsic pathways. Theivanthiran et al. (205) demonstrated that PD-1 blockade on CTLs in a syngeneic mouse model was able to activate a PD-L1-NLRP3 inflammasome signaling pathway in tumor cells that promoted MDSC recruitment and infiltration into the tumor microenvironment. Intratumoural MDSCs can suppress T cell function (193) and thus may dampen the immune response and promote resistance to anti-PD-1 therapies. The effect of the immunotherapy drug Atezolizumab was measured on MDA-MB-231 breast cancer cells (206). In this study, RNA-Seq was utilized to assess the modulation of gene expression after treatment with Atezolizumab and it was reported that genes promoting cell migration, metastasis, EMT, cell growth, and hypoxia were downregulated whilst apoptosis genes were upregulated. This suggests that Atezolizumab may be able to modulate the signaling of PD-L1 in this cell line to some extent at the level of gene expression. In contrast, Wang et al. (15), investigated the effects of anti-PD-1 antibodies Nivolumab and Pembrolizumab or the anti-PD-L1 antibody Atezolizumab on Calu-1, SW480, HT-29, BxPC-3, SK-BR-3, and U-2 OS cells. All immunotherapy drugs were shown to increase cell proliferation compared to isotype control *in vitro*. To verify these findings *in vivo*, human lung cancer cells were inoculated into immunocompromised mice. Similar to *in vitro* studies, monoclonal antibody administration to block PD-1 or PD-L1 activated PI3K and MAPK pathways by phosphorylating AKT and ERK1/2, respectively, promoting tumor cell growth *in vivo*. These small numbers of studies suggests that immunotherapeutic antibodies may be able to modulate the intrinsic function of PD-L1 and PD-1 and potentially highlights another mechanism by which tumors may develop resistance to PD-1/PD-L1 targeting therapy through co-expressing PD-L1 and its receptor PD-1. The ability of immunotherapy drugs to modulate the intrinsic PD-L1 and PD-1 pathway in other cancers in more heterogeneous tumor models could also provide further important insight into the mechanism of immunotherapy treatment.

## Future Direction “Modeling Tumor Heterogeneity” to Further Elucidate Intrinsic Roles of PD-L1 and PD-1

Tumor heterogeneity makes it challenging to identify novel therapeutic targets and potential biomarkers of immunotherapy response that could substantially enhance therapeutic efficacy. The scientific basis for numerous clinical trials has derived from 2D cell culture models and animal models, which can fail to fully replicate the human tumor microenvironment due to lack of heterogeneity and species-to-species variability, respectively, which could account for lack of transferability of PD-1/PD-L1-targeted antibodies into the clinic (24, 204). Furthermore, most evidence to date exploring the intrinsic role of PD-L1 and PD-1 has been based on 2D cell culture models using murine or human cancer cell lines or animal models, and thus limit the capacity to explore these roles in a relevant human tumor setting. Given the emerging intrinsic roles of PD-L1 and PD-1 and the differences between cancer types, utilizing models which closely mimic the heterogeneity of the human tumor microenvironment could allow a more predictive *in vitro* evaluation of the intrinsic role of PD-L1 and PD-1 in cancer and modulation by anti-cancer therapeutics. For example, human cancer cells implemented into different 3D cell culture models have shown to exhibit characteristics that more closely mimic *in vivo* human tumors, such as changes in morphology, proliferation, gene and protein expression, and response to treatment (204). Indeed the modulation of PD-L1 expression has been reported to be affected by the extracellular matrix stiffness of tumors in 3D culture (207) and a 3D model system utilizing patient-derived organoids that resembled the tumor immune microenvironment for the study of the PD-1/PD-L1 signaling axis has been developed (208). Furthermore, in a recent study a tumor-immune co-culture was utilized to assess the efficacy of immunotherapies Nivolumab and Durvalumab (209).

## Conclusions

PD-1/PD-L1 checkpoint blockade is at the cutting edge of research offering cancer patients hope for new treatment regimens with potential to have substantial clinical benefit and prolong survival. PD-1/PD-L1-targeted therapies reactivate the immune system to induce immune-mediated tumor eradication, and although they have demonstrated success has single agents, they have also shown cooperation with conventional and targeted therapies in the clinic. Unfortunately, most patients are unresponsive or develop resistance to PD-1/PD-L1-targeted therapy. Further elucidating the tumor intrinsic role of PD-L1 and its receptor PD-1 in all cancer types will help understand the basis for or lack of response to immunotherapy and may allow the identification of novel therapeutic targets and biomarkers to enhance clinical efficacy.

## Author Contributions

KH and RL researched and wrote the manuscript. KH designed the figures. RL conceived the theme/direction. NC and NJ-M reviewed the manuscript. All authors contributed to the article and approved the submitted version.

## Conflict of Interest

The authors declare that the research was conducted in the absence of any commercial or financial relationships that could be construed as a potential conflict of interest.

## Abbreviations

NK: Natural killer cells
Tregs: Regulatory T cells
MDSCs: Myeloid-derived suppressor cells
PD-L1: Programmed death-ligand 1
PD-1: Programmed death 1
FDA: USA Food and Drug Administration
NSCLC: Non-small cell lung cancer
CTLA-4: Cytotoxic T lymphocyte antigen-4
TCR: T cell receptor
APCs: Antigen presenting cells
PD-L2: Programmed death-ligand 2
SHP1/2: Src-homology 2 domain-containing phosphatase 1/2
miRNA: microRNA
3'UTR: 3&#8217
untranslated region
GSK3β: Glycogen synthase kinase 3β
S/T: Serine/Threonine
ccRCC: Clear cell renal cell carcinoma
ER: Endoplasmic reticulum
2D: Two-dimensional
CSC: Cancer stem cell
EMT: Epithelial to mesenchymal transition
3D: Three-dimensional
MDR1/P-gp: Multidrug resistant 1/P-glycoprotein.
